# Correlation Between Interatrial Septal In Situ Thrombus and Stroke Evaluated by Transesophageal Echocardiography

**DOI:** 10.31083/RCM50472

**Published:** 2026-07-24

**Authors:** Bin Wang, Shuang Wang, Minxin Zhang, Kehan Ding, Shuixing Zhang

**Affiliations:** ^1^Department of Radiology, The First Affiliated Hospital of Jinan University, 510627 Guangzhou, Guangdong, China; ^2^Department of Cardiovascular Ultrasound, Zhongnan Hospital of Wuhan University, 430071 Wuhan, Hubei, China

**Keywords:** patent foramen ovale, stroke, migraine, transesophageal echocardiography, *in situ* thrombus, c-TEE

## Abstract

**Background:**

Patent foramen ovale (PFO) is a crucial etiology of cryptogenic stroke, with paradoxical embolism recognized as its classic pathogenic mechanism. However, the origin of most emboli remains unclear. This study aimed to detect the presence and location of *in situ* thrombus on the interatrial septum in patients with PFO using contrast-enhanced transesophageal echocardiography (c-TEE) and analyze its correlation with stroke and migraine, thereby providing novel insights into the pathogenesis of PFO-related diseases.

**Methods:**

A retrospective analysis was performed on 3343 patients who underwent TEE examination between March 2021 and December 2024. Patients were divided into the *in situ* thrombus group (n = 62) and the non-*in situ* thrombus group (n = 1211) based on TEE findings. According to clinical symptoms, patients were categorized into the stroke group (n = 210), the migraine group (n = 633), and the asymptomatic group (n = 430). TEE was used to comprehensively assess the location, size, and quantity of the *in situ* thrombus. The anatomical parameters and hemodynamic parameters of PFO were compared among different groups.

**Results:**

Among 1273 PFO patients, the overall detection rate of *in situ* thrombus on the interatrial septum by TEE was 4.56% (62/1273). The detection rate of *in situ* thrombus in the stroke group (11.90%, 25/210) was significantly higher than that in the migraine group (2.53%, 16/633) and the asymptomatic group (3.95%, 17/430) (*p *< 0.001). In situ thrombus was most commonly located on the right atrial surface of the interatrial septum (69.0%, 40/62). The PFO diameter in the thrombus group was significantly larger than that in the non-thrombus group (*p *= 0.0033), and the shunt velocity was also significantly higher (*p *< 0.001). At the same time, no significant difference in PFO length was observed between the two groups. All patients with a detected *in situ* thrombus had negative findings on lower-extremity venous ultrasonography. After PFO closure, the rate of symptom improvement in the stroke group was significantly higher than in the migraine group (95.68% vs 52.80%, *p *< 0.005).

**Conclusions:**

c-TEE is an effective method for detecting *in-situ* thrombus in the atrial septum in patients with PFO, and such thrombus may play an important role in PFO-related stroke. PFO closure may be effective in stroke patients with concomitant *in-situ* thrombus. In clinical practice, routine risk stratification should be performed in patients with PFO, with an emphasis on identifying high-risk features and *in situ* thrombus lesions, to provide a basis for individualized treatment decisions.

## 1. Introduction

A patent foramen ovale (PFO) is a congenital cardiac structural abnormality characterized by the persistent patency of the physiological foramen ovale in the interatrial septum after birth, due to its failure to close normally. The autopsy detection rate of PFO in the general population is approximately 27.3% [1]. PFO is currently recognized to be associated with a variety of diseases, including cryptogenic ischemic stroke, transient ischemic attack (TIA), migraine, and peripheral or coronary artery embolism [2,3,4,5]. Cryptogenic stroke attributable to PFO accounts for about 5% of all ischemic strokes, and this proportion can be as high as 10% in young and middle-aged populations [5]. Therefore, identifying high-risk PFO patients who may benefit from antithrombotic therapy or interventional closure is clinically important [6,7,8,9,10,11,12,13,14,15,16,17,18].

Although the clinical relevance of PFO has been widely recognized, identifying pathogenic PFO remains highly challenging due to the lack of reliable predictive indicators. The detection rate of deep vein thrombosis in patients with PFO-related stroke is only approximately 10% [19,20,21,22,23,24], suggesting that the embolic source is still unclear in the vast majority of patients. In addition to serving as a conduit for paradoxical embolism from deep vein thrombosis, recent studies have demonstrated that *in situ* thrombosis within the PFO may also contribute to ischemic stroke [25,26,27]. Furthermore, accumulating evidence regarding atrial arrhythmias and the accumulation of small-molecule metabolites has provided new perspectives for elucidating the pathogenesis of PFO-related stroke [28,29,30,31]. The obstruction of cerebral blood vessels by emboli and the subsequent impairment of brain function in the corresponding regions constitute the core pathophysiological process of stroke [32]. Therefore, identifying the source of the causative emboli is of great clinical significance. We believe that the current understanding of PFO-related stroke is still limited, and further exploration of its novel pathogenic mechanisms and potential therapeutic targets is urgently needed.

Transesophageal echocardiography (TEE) enables accurate evaluation of intracardiac structures and functional characteristics, serving as the gold standard and first-line imaging modality for the diagnosis of PFO. It also represents one of the most advanced techniques currently available for identifying potential cardiogenic emboli in stroke [33,34]. Compared with TTE, TEE offers significant advantages in detecting structural cardiac abnormalities, including valve prolapse, intracardiac vegetations, and thrombi in the left atrium and left atrial appendage [35,36]. In our clinical practice, we successfully identified *in situ* thrombi on the right side of the interatrial septum using TEE. Motivated by this clinical observation, the present study was designed to conduct a systematic investigation. We aimed to characterize the location, size, and morphology of microthrombi and preliminarily analyze their correlation with stroke incidence. In addition, we incorporated clinical and laboratory parameters to explore potential risk factors underlying microthrombus formation, with the ultimate goal of providing novel scientific evidence for the development of preventive and therapeutic strategies for PFO-related stroke. We proposed the following hypotheses: (1) *In situ* microthrombi on the right interatrial septum represent a distinct subtype of right atrial thrombus; (2) Their formation may be closely associated with the presence of PFO; (3) Such microthrombi may constitute a novel potential etiology of PFO-related stroke.

## 2. Materials and Methods

### 2.1 Study Subjects

A retrospective analysis was performed on 3343 patients who underwent TEE at our hospital from March 2021 to December 2024. The inclusion and exclusion criteria of this study were as follows.

#### 2.1.1 Inclusion Criteria

(1) Patients diagnosed with PFO by TTE, contrast-enhanced transthoracic echocardiography (c-TTE), TEE, and contrast-enhanced transesophageal echocardiography (c-TEE).

(2) Patients who were hospitalized in our hospital and had completed imaging examinations and clinical data collection.

(3) All patients or their family members signed informed consent before undergoing TEE examination.

#### 2.1.2 Exclusion Criteria

(1) Patients with atrial septal defect or complicated with other severe congenital heart diseases, valvular diseases, cardiomyopathy, etc., were excluded by TTE and TEE.

(2) Patients complicated with various critical illnesses such as sepsis and cerebral hemorrhage.

(3) Patients complicated with various malignant tumors, liver and renal failure, etc.

(4) Patients with hematological and hemorrhagic diseases.

(5) Patients with oral or esophageal diseases who were unable to undergo intubation for TEE examination.

(6) Patients with mental disorders who were unable to cooperate with the examination.

(7) Patients with incomplete clinical data.

**Fig. 1.**
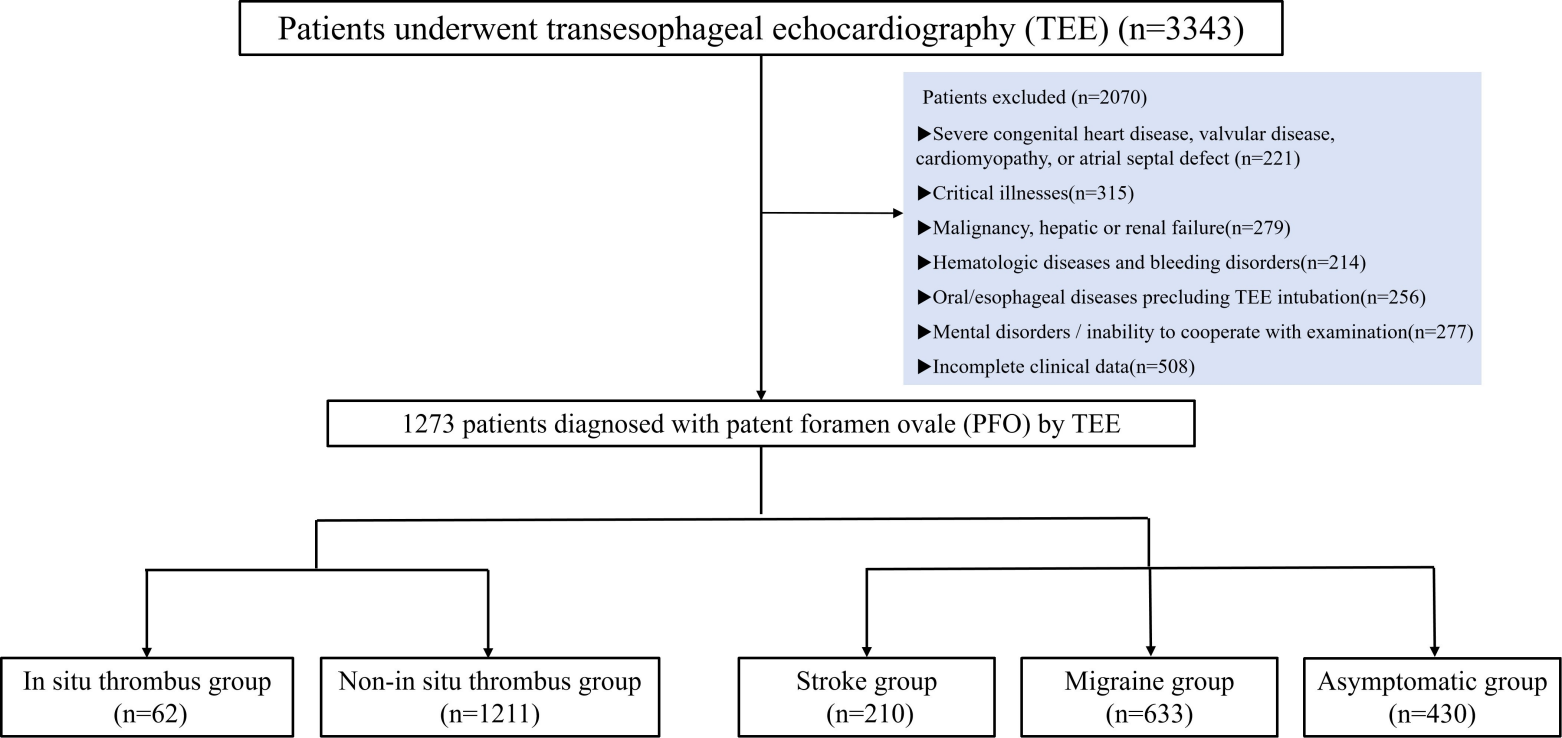
**Flowchart of inclusion and exclusion criteria for subjects and group allocation**.

A total of 1273 patients diagnosed with PFO were finally included in the study. Patients were divided into the *in-situ* thrombus group (n = 62) and the non-*in situ* thrombus group (n = 1211) based on TEE findings. Based on the clinical symptoms, patients were categorized into the stroke group (n = 210), the migraine group (n = 633), and the asymptomatic group (n = 430) (Fig. 1).

### 2.2 Instruments and Methods

#### 2.2.1 Instruments

Philips EPIQ CVx and EPIQ 7C ultrasound diagnostic systems were used. The transthoracic two-dimensional probe was S5-1 with a frequency of 2.0–4.5 MHz and a frame rate of 50–70 frames per second. The transesophageal phased-array volume probes were X8-2t and X7-2t, with a frequency range of 3.0–8.0 MHz.

#### 2.2.2 Image Acquisition of c-TTE

During the examination, all patients were placed in the left lateral decubitus position with their arms placed above their heads, and an electrocardiograph was connected. All patients underwent TTE before TEE to clarify cardiac and valvular conditions and to exclude other structural abnormalities. For right heart contrast-enhanced imaging, the apical four-chamber view was used for observation, and dynamic images of 20 cardiac cycles were stored while the patients maintained normal breathing.

Preparation Procedure of Contrast Agent for c-TTE [37]: An 18-G indwelling needle was inserted into the median cubital vein of the patient’s left arm and connected to a three-way stopcock. Two 10 mL syringes were attached to either end of the three-way stopcock. One syringe was filled with 8 mL of 0.9% sodium chloride solution and then connected to the three-way stopcock. Following this, 1 mL of the patient's venous blood was aspirated back into the syringe from the indwelling needle. The other syringe was filled with 1 mL of air and connected to the three-way stopcock. The two syringes were rapidly pushed and oscillated back and forth about 10 times to generate a large number of microbubbles. In a resting state, the microbubbles were rapidly injected in a "bolus" manner, and images were recorded. Observation was performed from the opacification of the right atrium until the contrast agent in the cardiac chambers disappeared. After a 1–2-minute interval, a second contrast-enhanced imaging of the right heart was performed with the patient performing the Valsalva maneuver. The steps were as follows: the patient took a deep breath and held it, then the microbubbles were rapidly injected as a "bolus". When the right atrium was filled with microbubbles, the patient was instructed to exhale rapidly. Dynamic images of 20 cardiac cycles were immediately stored at the start of injection. Then the images were played back frame by frame to select the frame with the maximum number of microbubbles in the left atrium within 3–5 cardiac cycles for counting. Before the Valsalva maneuver examination, the operator explained the key points of the Valsalva maneuver to the patient (instructing the patient to hold their breath at the end of deep inspiration, then relax and exhale rapidly, or to cough continuously and rapidly) and provided training. The operator judged the patient's cooperation based on the patient's condition, thoracic movement, the degree of lung coverage of the heart, and changes in abdominal wall tension.

#### 2.2.3 Image Acquisition of c-TEE

Before transesophageal echocardiography, all patients were fasted for 8 hours, and infectious disease screening was negative (the probe and examination room were strictly disinfected in accordance with hospital infection control requirements after the procedure). Preoperative informed consent was obtained, and an indwelling needle was inserted into the cubital vein of the left upper extremity. The patient was placed in the left lateral decubitus position, and local anesthesia was performed by gargling with lidocaine gel. A bite block was placed, and the transesophageal probe was inserted into the middle esophagus (approximately 35 cm from the incisor teeth) to observe the interatrial septum. Two-dimensional, color Doppler, and biplane scanning were performed continuously from 0° to 180° to fully display the fossa ovalis of the interatrial septum in the resting state. Special attention was paid to observing the adhesion between the primary septum and the secondary septum in the middle esophagus (70°–120°), the presence of gaps, the presence of transseptal blood flow by color Doppler, and the presence of *in situ* thrombus on the left atrial surface of the interatrial septum, the right atrial surface of the interatrial septum, and within the fossa ovalis. The size, quantity, and mobility of *in situ* thrombus were measured, and dynamic images of 20 cardiac cycles were stored. The patient was instructed to cooperate with the Valsalva maneuver (taking a deep breath and then forcefully bulging the abdomen; if the patient was unable to exert force, an assistant could press the patient's abdomen to assist). The interatrial septum was observed to lift toward the left atrial side, and dynamic images of 20 cardiac cycles were stored. For patients with PFO, the blood flow velocity at the PFO shunt was measured by adjusting the angle in the mid-esophageal or gastric fundus view. Then, right heart contrast-enhanced imaging was performed under TEE using the same method as c-TTE. The c-TEE examination was performed three times, and images were stored each time. The first and second examinations were performed in the resting state and during the Valsalva maneuver, respectively, to observe the presence of right-to-left shunt (RLS) at the interatrial septum level. The third examination was performed by rotating the probe to 45°–90° to display the long axis of the left upper pulmonary vein for observation. An interval of 1–2 minutes was maintained between each examination until the microbubbles in the cardiac chambers disappeared from the previous examination.

The standard for good cooperation with the Valsalva maneuver was as follows: large amplitude of interatrial septum movement, sufficient opening of the foramen ovale, obvious RLS, and significant passage of contrast microbubbles through the foramen ovale to the left atrium.

### 2.3 Image Analysis

**Fig. 2.**
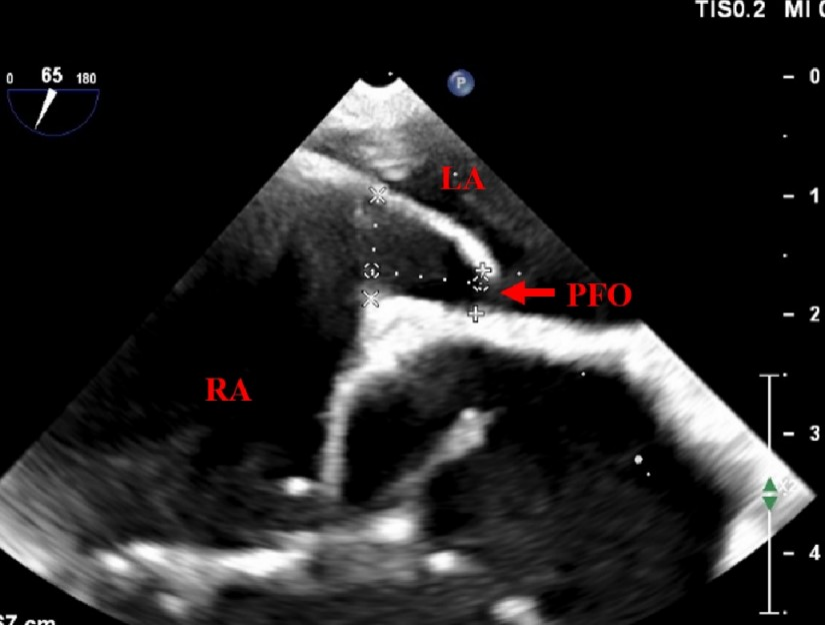
**Schematic diagram of PFO size measurement by TEE (LA: left atrium; RA: right atrium)**. PFO, patent foramen ovale; TEE, transesophageal echocardiography.

**Fig. 3.**
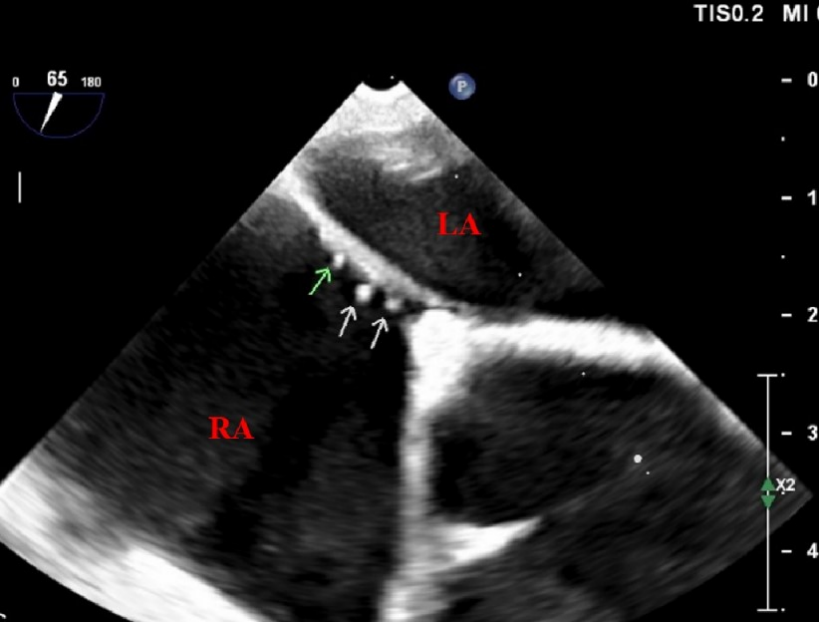
**TEE showing multiple microthrombi on the right atrial surface of the interatrial septum (indicated by arrows, LA: left atrium; RA: right atrium)**.

The TEE images stored were played back frame by frame by the image analysis operator. The maximum gap width between the primary septum and the secondary septum was routinely measured as the PFO diameter, and the maximum overlapping length between the primary septum and the secondary septum was measured as the PFO length. The size, quantity, and mobility of microthrombi were measured as key parameters (Figs. 2,3).

In c-TEE mode, images were played back frame by frame to observe the number and flow of contrast agent microbubbles entering the left atrium through the PFO after right atrial opacification, as well as the PFO volume. RLS was recorded. Meanwhile, the volume of contrast agent microbubbles entering the left atrium through the long axis of the left upper pulmonary vein was recorded. RLS grading criteria based on microbubble count [37]:

Grade 0: no microbubbles in the left cardiac chambers, indicating no RLS.

Grade Ⅰ: less than 10 microbubbles visible in the left cardiac chambers, indicating mild RLS.

Grade Ⅱ: 10–30 microbubbles in the left cardiac chambers, indicating moderate RLS.

Grade Ⅲ: more than 30 microbubbles visible in the left cardiac chambers, indicating severe RLS.

The final result was determined based on the frame with the highest number of microbubbles in the PFO and the left upper pulmonary vein.

### 2.4 Statistical Analysis

All statistical analyses were performed using GraphPad Prism software (version 9.0, GraphPad Prism Software Inc., San Diego, CA). Continuous variables were expressed as mean ± standard deviation (Mean ± SD), and categorical variables were described as frequency (percentage). Intergroup comparisons were performed using appropriate methods based on data type and distribution characteristics. For continuous variables conforming to a normal distribution, an independent-samples *t*-test (for two groups) or a one-way analysis of variance (ANOVA) (for multiple groups) was used to compare differences. For continuous variables not satisfying the normal distribution, the Wilcoxon rank-sum test was used. Correlation analysis between categorical variables was performed using the chi-square test or Fisher's exact test, depending on expected frequencies. A* p*-value < 0.05 was considered statistically significant for all hypothesis tests.

## 3. Results

### 3.1 Basic Characteristics

Analysis of microthrombi detection in patients with different symptoms showed significant differences among the groups. The stroke group had 25 patients with detected microthrombi, with a detection rate of 11.90%. 16 cases were detected in the migraine group, with a detection rate of 2.53%. 17 cases were detected in the asymptomatic group, with a detection rate of 3.95%. The above results indicated that the detection rate of microthrombi in stroke patients was significantly higher than that in migraine patients and asymptomatic individuals, suggesting that microthrombi formation may be associated with the pathogenesis of stroke (Table 1).

**Table 1.** **Comparison of microthrombi detection in patients with different clinical symptoms**.

General characteristics	Stroke group	Migraine group	Asymptomatic group	F/χ^2^ value	*p* value
	(n = 210)	(n = 633)	(n = 430)		
Microthrombi (n, %)	29 (13.81%)^bd^	16 (2.53%)	17 (3.95%)	44.497	<0.001
Age (Mean ± SD, years)	53.31 ± 13.33^a^	46.78 ± 15.89^d^	56.58 ± 16.68	54.221	<0.001
Gender (Male, %)	126 (60.00%)^bc^	223 (35.23%)^c^	213 (49.53%)	46.883	<0.001

Note: compared with the migraine group, ^a^*p* < 0.05, ^b^*p* < 0.001; compared with the asymptomatic group, ^c^*p* < 0.05, ^d^*p* < 0.001. SD, standard deviation.

There were significant differences in the detection rate of microthrombi among the three groups (*p *< 0.001). The detection rate was the highest in the stroke group (11.90%, 25/210), followed by the asymptomatic group (3.95%, 17/430), and the lowest in the migraine group (2.53%, 16/633). There were also significant differences in age among the three groups (overall *p *< 0.001). Pairwise comparisons showed that the migraine group was significantly younger (46.78 ± 15.89 years) than the stroke group (53.31 ± 13.33 years, *p *< 0.001) and the asymptomatic group (56.58 ± 16.68 years, *p *< 0.001). The asymptomatic group was slightly older than the stroke group (*p *= 0.008). There were also significant differences in gender composition (*p *< 0.001). The stroke group had a higher proportion of males (60.00%). The migraine group was predominantly female (64.77%). The asymptomatic group had a nearly balanced gender ratio (49.53% males). Overall, the results suggested that microthrombi were more common in stroke patients, while the migraine population was younger and had a higher proportion of females.

### 3.2 Analysis of Microthrombi Location Characteristics and Group Comparison in Stroke and Migraine Groups

**Fig. 4.**
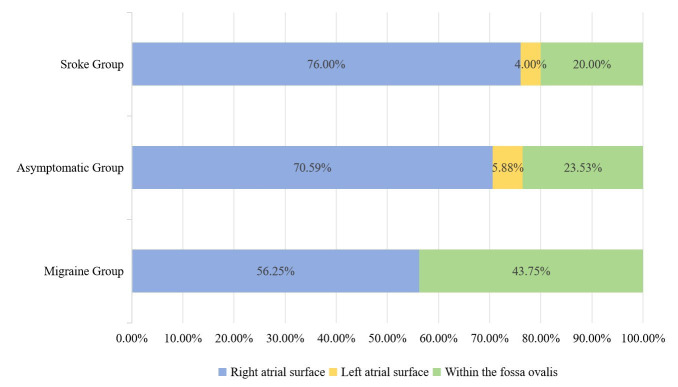
**Percentage stacked bar chart of location characteristics and grouping of microthrombi**.

Statistical analysis showed no significant differences in multiple indicators of thrombus characteristics between the stroke and migraine groups. Specifically, the Wilcoxon rank-sum test was used to compare thrombus size (*p *= 0.2732) and thrombus quantity (*p *= 0.5439) between the two groups; neither difference was statistically significant (*p *> 0.05). Further comparison of thrombus location classification using Fisher's exact test yielded a *p*-value of 1, indicating no significant bias in the distribution of thrombus location between the two groups. Due to the small sample size, the statistical power may be insufficient, which may explain the conclusion of "no significant difference". In summary, in the study cohort, the size, quantity, and location characteristics of thrombus were not significant factors distinguishing stroke patients from migraine patients (Table 2 and Fig. 4).

**Table 2.** **Location characteristics of microthrombi and comparison between different groups**.

Location	Stroke group	Migraine group	Asymptomatic group	χ^2^ value	*p* value
Right atrial surface of the IAS	21 (77.78%)	10 (58.82%)	13 (72.22%)	0.53	0.766
Left atrial surface of the IAS	1 (3.70%)	0 (0.00%)	1 (5.56%)	0.87	0.647
Within the fossa ovalis	5 (18.52%)	7 (41.18%)	4 (22.22%)	2.20	0.333
Total (n, %)	27 (43.55%)	17 (27.42%)	18 (29.03%)	3.61	0.462

Note: IAS, Interatrial septum; Fisher’s exact test was employed*.*

According to different thrombus locations, the number of microthrombi on the left atrial surface of the interatrial septum (IAS), the right atrial surface of the IAS, and within the fossa ovalis was counted in the three groups. The results showed that the distribution of microthrombi in the three groups was mainly on the right atrial surface of the IAS, accounting for 76% (stroke group), 56.25% (migraine group), and 70.59% (asymptomatic group), respectively. The proportions of thrombi on the left atrial surface of the IAS and within the fossa ovalis were relatively small. The overall comparison showed no significant difference in the distribution of microthrombi among the three groups (*p *= 0.366) (Table 2 and Fig. 4).

### 3.3 Comparison of Symptom Improvement in Stroke and Migraine Patients After Closure

Analysis of symptom improvement following closure in patients with stroke and migraine showed significant differences in the distribution of postoperative outcomes between the two groups (*p *< 0.005). In the stroke group (n = 139), the vast majority of patients experienced symptom improvement after surgery (133 cases, 95.68%), while only a small proportion showed only a slight symptom improvement (5 cases, 3.60%) or no improvement (1 case, 0.72%). In contrast, in the migraine group (n = 339), 179 cases (52.80%) showed improved symptoms, 74 cases (21.83%) showed slight improvement, and 86 cases (25.37%) showed no improvement (see Table 3).

**Table 3.** **Comparison of the improvement of symptoms in stroke and migraine patients after closure**.

Postoperative symptoms	Stroke group	Migraine group	χ^2^ value	*p* value
Improved (n, %)	133 (95.68%)	179 (52.80%)	82.18	<0.005
Slightly improved (n, %)	5 (3.60%)	74 (21.83%)	26.92	<0.005
Unimproved (n, %)	1 (0.72%)	86 (25.37%)	36.24	<0.005
Total (n, %)	139 (100%)	339 (100%)	82.18	<0.005

Note: Improved: disappearance of stroke and migraine symptoms half a year after surgery; Slightly improved: reduction in the frequency of stroke and migraine attacks compared with before surgery; Unimproved: no reduction in the frequency of stroke and migraine attacks compared with before surgery.

### 3.4 Comparison of PFO Characteristics and Shunt Velocity Between the Thrombus Group and the Non-Thrombus Group

Nonparametric tests were used to compare the differences in PFO morphological and hemodynamic parameters between the thrombus group and the non-thrombus group. Statistical analysis showed that the PFO diameter in the thrombus group was significantly larger than that in the non-thrombus group (*p *= 0.0033). There was no significant difference in PFO length between the two groups (*p* = 0.1258). The shunt velocity in the thrombus group was significantly higher than that in the non-thrombus group (*p *< 0.001). The above results suggest that larger PFO diameter and higher shunt velocity may be associated with thrombus formation, whereas PFO length is not a key factor affecting thrombus formation.

## 4. Discussion

**Fig. 5.**
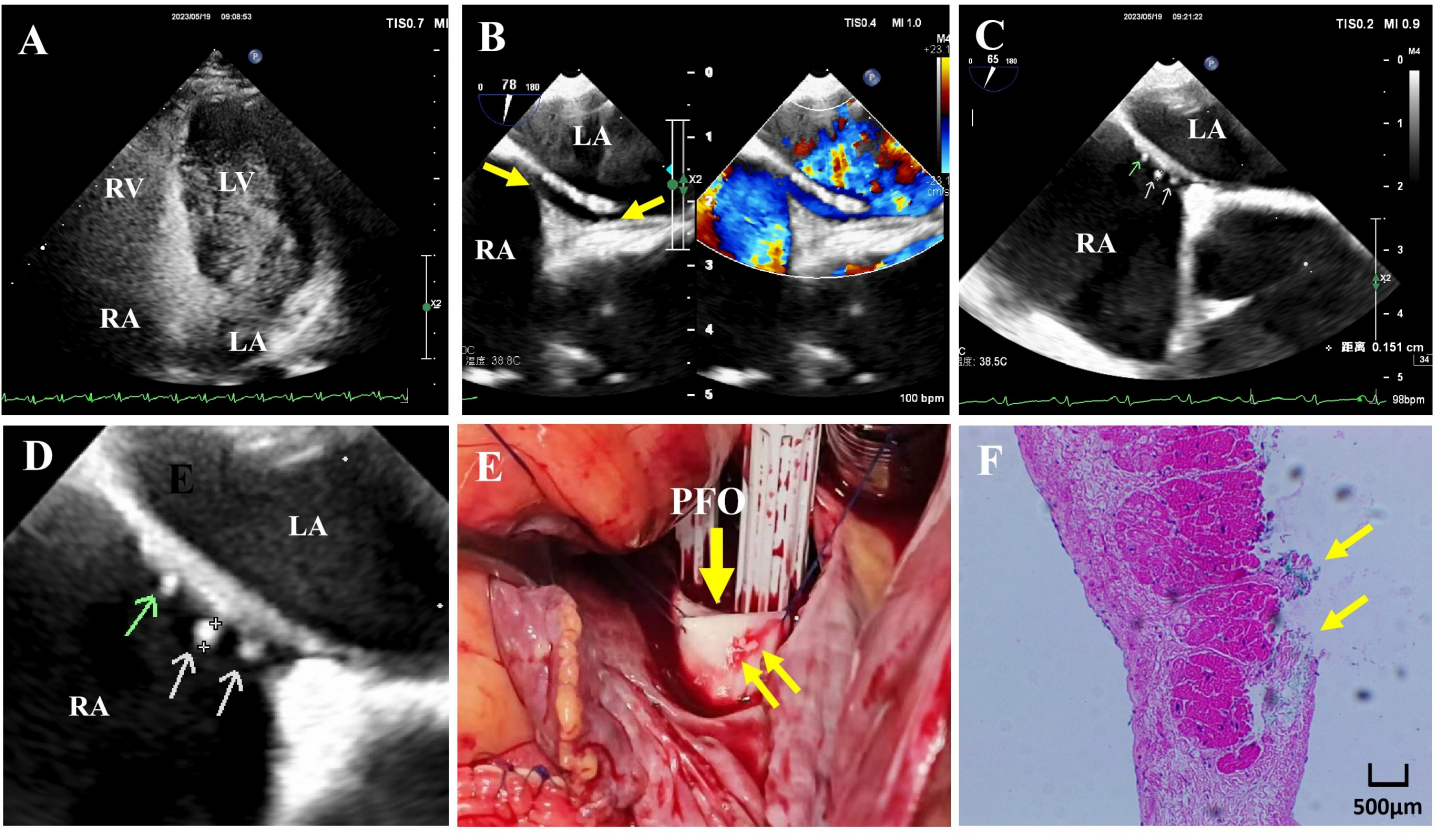
**Typical case of *in situ* microthrombus in the atrial septum**. (A) Saline Contrast Echocardiography showing an extremely large intracardiac right-to-left shunt (RLS) after the Valsalva maneuver. (B) Transesophageal echocardiography reveals a large patent foramen ovale (yellow arrows). (C,D) Transesophageal echocardiography displaying multi-tiny *in situ* thrombosis in the right atrial surface (green arrow and white arrows). (E) Intraoperative photo displaying the patent foramen ovale and *in situ* thrombosis (yellow arrows). (F) Pathological analysis verifying the existence of *in situ* thrombosis (yellow arrows), Scale bar = 500 μm. LA indicates left atrium; LV, left ventricle; RA, right atrium; RV, right ventricle; and PFO, Patent foramen ovale.

**Fig. 6.**
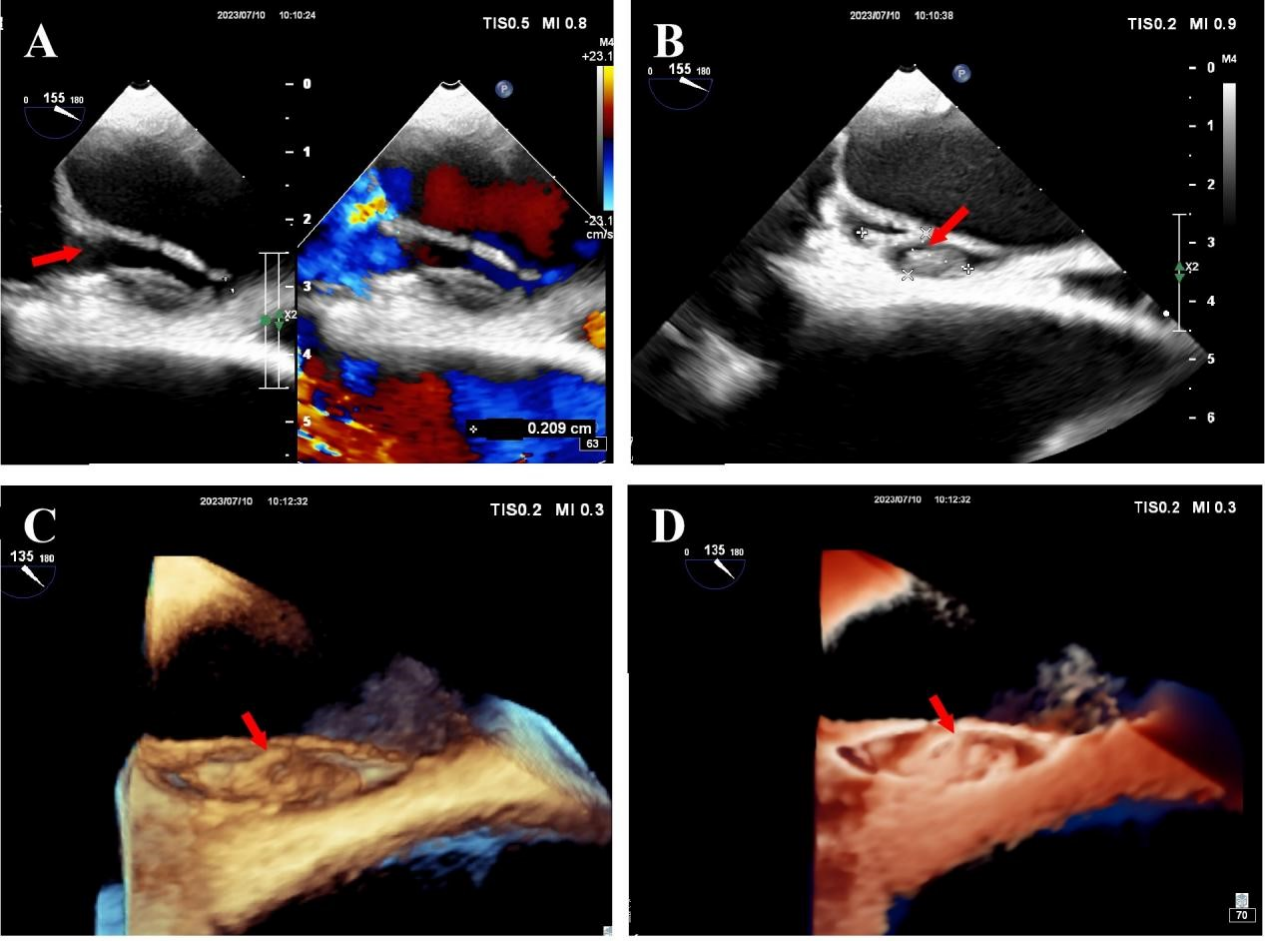
**Schematic diagram of thrombus within an aneurysmal PFO**. (A) The TEE shows an unclosed channel within the fossa ovalis, with blood flow signals passing through the PFO from the right atrial surface of the interatrial septum (red arrows) to the left atrium (LA). (B) Two-dimensional TEE showing hypoechoic lesions of thrombus within the aneurysmal PFO (red arrows). (C,D) Three-dimensional transesophageal echocardiography (3D-TEE) showing hypoechoic lesions of thrombus within the aneurysmal PFO under different modes (red arrows).

**Fig. 7.**
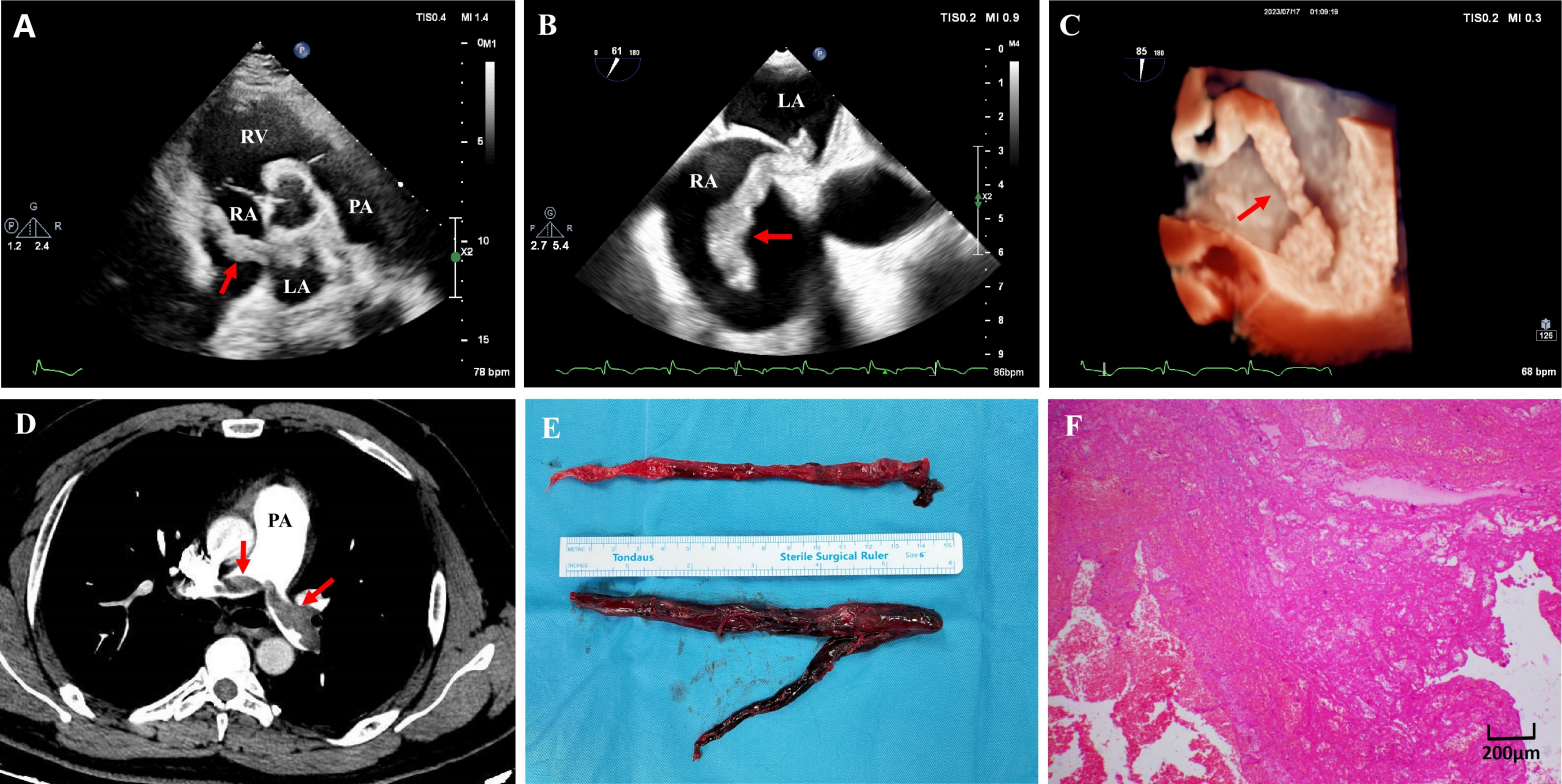
**Schematic diagram of serpentine straddling thrombus within PFO**. (A) TTE showing striped hypoechoic lesions (red arrows) passing through the PFO from the right atrium (RA) to the left atrium (LA). (B,C) TEE and three-dimensional transesophageal echocardiography (3D-TEE) showing complex serpentine thrombi (red arrows) continuously extending from the RA to the LA through the PFO. (D) Pulmonary computed tomography angiography (CTA) showing fresh thrombi in the main pulmonary artery (PA) and its branches (red arrows). (E) During surgery, a solid thrombus approximately 15 × 1 cm in size was resected from the fossa ovalis, along with a Y-shaped thrombus originating from the main PA and its branches. (F) Pathological results confirmed that the surgical specimen was a thrombus, Scale bar = 200 μm. This image is sourced from Ref. [42]. LA indicates left atrium; PA, Pulmonary artery; RA, right atrium; RV, right ventricle; and PFO, Patent foramen ovale.

PFO is a significant risk factor for cryptogenic stroke, and its underlying pathogenic mechanisms have long been a major focus of research in the fields of cardiology and neurology. Although the classic paradoxical embolism theory has been widely recognized, deep vein thrombosis can only be detected in approximately 10% of PFO-related stroke patients in clinical practice [38,39], indicating the existence of incompletely elucidated pathogenic pathways. In recent years, the discovery of *in situ* thrombus formation around the PFO tunnel has provided a new perspective on this clinical phenomenon, and the utility of transesophageal echocardiography (TEE), the gold standard for PFO diagnosis, in identifying *in situ* thrombus has gradually attracted attention [40,41]. PFO-related *in situ* thrombus is defined as a thrombus that forms directly via local blood coagulation and adheres to the endocardial surface within the PFO channel, rather than an embolus that detaches from the venous system and migrates through the PFO channel. Yan et al. [26] investigated the association between stroke and *in situ* thrombus using high-resolution optical coherence tomography (OCT) and found a significant correlation between *in situ* thrombus and stroke risk; *in situ* thrombus was also detected in migraine patients. These findings suggest that *in situ* thrombus may play an important role in PFO-related stroke or migraine patients. In our previous clinical practice, we identified different morphological types of thrombosis in the vicinity of the interatrial septum (including within the PFO tunnel), such as right atrial septal microthrombi, thrombi within the PFO tunnel, and serpentine thrombi straddling the PFO (see Fig. 5,6,7), and have reported relevant case studies [42]. Based on preliminary observations, this study collected TEE examination data from a large sample of PFO patients, systematically analyzed the detection characteristics, related influencing factors, and clinical correlations with stroke and migraine of interatrial septal *in situ* thrombus, aiming to further improve the pathogenic mechanism system of PFO-related diseases and provide evidence for clinical risk stratification and treatment decision-making.

In this study, the overall detection rate of interatrial septal *in situ* thrombus was 4.56% among 1273 PFO patients, with a significantly higher detection rate in the stroke group (11.90%) than in the migraine group (2.53%) and the asymptomatic group (3.95%). Additionally, lower-extremity venous ultrasound results were negative in all patients with thrombus, suggesting that an *in situ* interatrial septal thrombus is another important source of embolism, independent of paradoxical embolism from lower-extremity deep vein thrombosis. In terms of thrombus distribution, the right atrial surface was the predominant site of *in situ* thrombus formation (69.0%), and this pattern did not significantly differ among the stroke, migraine, and asymptomatic groups, a result closely related to the anatomical and hemodynamic characteristics of the fossa ovalis. As the thinnest region of the interatrial septum, the fossa ovalis is located at the left upper side of the inferior vena cava orifice. Chronic effects of inferior vena cava regurgitation on blood flow are prone to cause local endocardial microinjury via wall shear stress; exposed collagen and tissue factors can activate the coagulation pathway, promote platelet and red blood cell adhesion and aggregation, and ultimately lead to microthrombus formation [43]. This also provides anatomical and pathophysiological evidence for the mechanism of *in situ* thrombus formation.

This study further analyzed the relationship between PFO anatomical and hemodynamic parameters and *in situ* thrombus formation. The results showed that the PFO diameter in the thrombus group was significantly larger than that in the non-thrombus group (*p *= 0.0033), and the shunt velocity was also significantly higher (*p* < 0.001). At the same time, no significant difference in PFO length was observed between the two groups. These findings indicate that a larger PFO diameter and higher shunt velocity are independent promoters of *in situ* thrombus formation in the interatrial septum. Although this result seems to contradict the classic theory that "blood stasis within the PFO promotes thrombus formation", it is highly consistent with the hemodynamic characteristics of the region adjacent to the PFO: the high shunt velocity associated with a large-diameter PFO exacerbates the impact injury of inferior vena cava blood flow on the right atrial surface of the fossa ovalis, and the vortex formed by high-velocity transseptal blood flow causes local hemodynamic disturbance, which not only induces endocardial injury but also provides dynamic conditions for platelet aggregation. These two factors jointly promote thrombus formation. The lack of association between PFO length and thrombus formation in this study may be related to the detection resolution of TEE. TEE has high sensitivity in detecting microthrombi on the right atrial side near the PFO. Still, it cannot identify micron-scale microthrombi within the PFO tunnel, which also explains the difference in detection results compared with OCT [25]. The overall detection rate of right atrial septal *in situ* thrombus by TEE in this study was 4.9%. The positional distribution of *in situ* thrombus in the three groups was dominated by the "right atrial surface", accounting for 77.78% in the stroke group, 58.82% in the migraine group, and 72.22% in the asymptomatic group, respectively; the proportions of the "left atrial surface" and "intra-fossa ovalis" were both small. Overall comparison showed no significant difference in positional composition among the three groups (*p *= 0.453). Unlike optical coherence tomography, the detection rate of thrombus within the PFO tunnel was relatively low, which may also be attributed to the resolution limitations of TEE.

Notably, OCT is difficult to adopt in routine clinical practice, whereas TEE is easy to perform and highly accessible clinically. It can effectively identify clinically significant *in situ* thrombus and high-risk PFO features, making it a more suitable routine detection method for risk stratification of PFO patients. In addition, this study found a contradiction between the statements that "low-velocity transseptal blood flow provides the basis for microthrombus formation" and "long-channel PFO is more prone to microthrombus formation" in existing literature. Although long-channel PFO is theoretically prone to blood stasis in clinical practice [44], no association was found between long-channel PFO and thrombus formation in this study. Possible reasons include a small sample size of long-channel PFO in the study cohort or the masking of its stasis effect by the dominant role of large-diameter vessels and high flow velocity. This conclusion still needs to be verified by large-scale studies.

In this study, the *in situ* thrombus detection rate in the migraine group was significantly lower than that in the stroke group. The symptom improvement rate of PFO closure in migraine patients (52.8%) was much lower than that in the stroke group (95.7%), suggesting that the association between *in situ* thrombus and migraine is much weaker than that between *in situ* thrombus and stroke. The currently accepted pathogenic mechanism of PFO-related migraine is that a RLS allows vasoactive substances metabolized in the pulmonary circulation to enter the systemic circulation, leading to abnormal cerebrovascular vasomotor function [43]. This study found no direct evidence for the involvement of interatrial septal *in situ* thromboembolism in the pathogenesis of migraine, and the low thrombus detection rate in the migraine group further supports this classic hypothesis. Therefore, *in situ* thrombus is not the main pathogenic factor of PFO-related migraine. In clinical practice, the assessment of PFO patients complicated by migraine should focus on the degree of shunt rather than the presence or absence of *in situ* thrombus.

This study also found that PFO closure has a significant therapeutic effect on stroke patients complicated with *in situ* thrombus, with 95.7% of patients achieving symptom improvement after surgery. In this study, "symptom improvement" refers to the disappearance of stroke symptoms 6 months after surgery, and "mild improvement" refers to a reduction in the frequency of stroke attacks. All patients who underwent closure surgery also received standardized basic treatments, such as antiplatelet therapy and circulatory support. Therefore, the recovery of stroke symptoms is the combined result of PFO closure and basic treatment, rather than the single effect of closure surgery. However, by occluding the PFO channel, closure surgery eliminates the anatomical basis for cerebral embolism caused by the detachment of *in situ* thrombus. It remains a key intervention measure to reduce the risk of recurrent stroke. This study is the first to clearly distinguish between interatrial septal *in situ* thrombus and "transit thrombus": the former refers to thrombus formed in the vicinity of the fossa ovalis, and the latter refers to thrombus that embeds in the PFO channel after venous thromboembolism (as shown in the Figure). The two differ in pathophysiological mechanisms, and thus their clinical management strategies should also be distinct. In this study, device closure combined with dual antiplatelet therapy was routinely used for PFO patients complicated with *in situ* thrombus to prevent thrombus formation. Transit thrombus has been confirmed to originate from the lower extremities; surgical thrombectomy is only reported in case studies and applies to critically ill patients with large transit thrombus in the PFO channel complicated with pulmonary embolism. This treatment process provides a referable practical protocol for clinical practice. No interatrial septal pouches were detected in this study, which may be attributed to the low incidence of this structure in the sample cohort or to differences in research focus. Its association with *in situ* thrombus and stroke still needs to be further explored in subsequent studies.

## 5. Limitations

First, this is a single-center retrospective study, which may introduce selection bias. In addition, some microthrombi were not detected due to the resolution limitation of TEE, and the extrapolation of the results needs to be verified by multicenter, prospective studies. Second, the diagnosis of interatrial septal *in situ* thrombus in this study was based solely on TEE imaging features and lacked large-sample histopathological verification. Subsequent studies can further clarify the nature and formation mechanism of thrombus by adding pathological evaluation of intraoperative thrombus specimens. Third, this study did not explore the efficacy of anticoagulant therapy for *in situ* thrombus in depth; comparison of the efficacy between closure surgery and anticoagulant therapy requires long-term follow-up studies and remains a key focus for future research on PFO-related thrombus.

## 6. Conclusions

This study confirms that TEE is an effective method for detecting *in situ* interatrial septal thrombus in patients with PFO, and that such thrombus may be an important independent pathogenic factor in PFO-related stroke. PFO closure combined with basic treatment has a significant therapeutic effect on stroke patients complicated with *in situ* thrombus and can be used as the preferred clinical intervention plan. In clinical practice, TEE should be routinely used for risk stratification of PFO patients, with a focus on identifying high-risk features and *in situ* thrombus lesions to inform individualized treatment decisions. Future research needs to conduct further multicenter prospective studies, integrate technologies such as OCT and pathological assessment to more deeply explore the mechanism of *in situ* thrombus formation, and compare the long-term efficacy of anticoagulant therapy and closure surgery to provide more comprehensive, evidence-based guidance for the prevention and treatment of PFO-related stroke.

## Data Availability

The dataset examined in this study is available uponreasonable request from the corresponding authors.
